# Regulation of FBXO4-mediated ICAM-1 protein stability in metastatic breast cancer

**DOI:** 10.18632/oncotarget.20912

**Published:** 2017-09-15

**Authors:** Jae-Hyeok Kang, Mi-Young Choi, Yan-Hong Cui, Neha Kaushik, Nizam Uddin, Ki-Chun Yoo, Min-Jung Kim, Su-Jae Lee

**Affiliations:** ^1^ Department of Life Science, Research Institute for Natural Sciences, Hanyang University, Seoul, Korea; ^2^ Centre of Excellence in Molecular Biology (CEMB), University of The Panjab, Lahore, Pakistan; ^3^ Laboratory of Radiation Exposure and Therapeutics, National Radiation Emergency Medical Center, Korea Institute of Radiological and Medical Sciences, Seoul, Korea

**Keywords:** intercellular adhesion molecule-1, E3 ligase, FBXO4, stability, metastatic breast cancer

## Abstract

Advanced or progressive cancers share common traits such as altered transcriptional modulation, genetic modification, and abnormal post-translational regulation. These processes influence protein stability and cellular activity. Intercellular adhesion molecule-1 (ICAM-1) is involved in the malignant progression of various human cancers, including breast, liver, renal, and pancreatic cancers, but protein stability has not been deal with in metastatic breast cancer. Additionally, the relevance of the stability maintenance of ICAM-1 protein remains obscure. Here, we identified a novel interaction of E3 ligase FBXO4 that is specifically presented to ICAM-1. To understand how FBXO4 modulates ICAM-1 stability, we investigated ICAM-1-overexpressing or knockdown metastatic/non-metastatic breast cancers. ICAM-1 was found to influence tumor progression and metastasis, whereas FBXO4 regulated aggressive tumorigenic conditions. These results demonstrate that FBXO4 is a major regulator of ICAM-1 stability and that alterations in the stability of ICAM-1 can influence therapeutic outcome in metastatic cancer.

## INTRODUCTION

In general, advanced or highly metastatic cancer acquires genetic modifications that confer the cancer cells with higher aggressive potential activity than non-metastatic cancers, and various molecules are differentially expressed in metastatic and non-metastatic cancers [1]. Maintenance of the stability and level of proteins involved in migration and invasion are essential for malignant progression of aggressive cancers [1, 2]. Cancer cells survive using control and activation mechanisms similar to those that maintain protein stability. In addition, protein stability and activity are regulated by transcriptional modulation, genetic modification, and multiple molecular events [1, 3]. Eventually, these processes confer metastatic ability to the cells and maintain the stability of critical proteins, allowing long-term survival of cancer cells and cancer progression. It was recently reported that the protein intercellular cell adhesion molecule-1 (ICAM-1) is an important factor for the maintenance of malignant potential of cancer and that the protein stability of is associated with tumor progression [4, 5]. However, the molecular importance of this protein stability of ICAM-1 remains obscure. ICAM-1 is a transmembrane molecule involved in many important processes and has recently been reported to be involved in tumor progression, abnormality, and malignant phenotypes [4–7]. Additionally, expression of ICAM-1 has been observed in various types of cancers and is associated with advanced cancer stages; malignant phenotypes of breast, lung, and prostate cancers; and resistance to chemotherapy [6–9]. Particularly, advanced or metastatic breast cancer has high mortality because of its high aggression and metastatic activity and availability of fewer therapeutic options than for non-metastatic subtypes [10, 11]. Abhishek et al. reported that overexpression of ICAM-1 in metastatic breast cancer becomes more pronounced with increasing malignant alterations, and this protein is expressed in various cell types, functioning in numerous important processes such as cell-cell interactions, cell signaling, transmigration, and proliferation, as well as in maintenance of cellular stability [12–14]. Thus, increased ICAM-1 stability might promote cancer progression and metastatic activity, making it a potential major regulatory mechanism in cancerous conditions. Therefore, to reduce the stability of proteins involved in cancer, it is important to understand the regulation and inhibitory mechanisms of these proteins in metastatic cancer.

Cells and tissues have internal protective mechanisms against tumorigenesis, and when abnormal proteins are formed, various tumor suppressive processes are activated, including ubiquitin mediated proteasomal degradation, immune activation, or activation of cell repair mechanisms or tumor-suppressive network signals. Stabilized or destabilized proteins are rapidly degraded via the ubiquitin-proteasome pathway [14–16], and proteasomal degradation of abnormal proteins is one of the mechanisms through which tumor suppressor act. Ubiquitin ligase is associated with many diseases including cancer and immunological disorders; its key mechanisms, regulators, and pathways have been widely investigated [14–18]. Ubiquitin-mediated proteasomal degradation is an irreversible mechanism utilized in various processes that are regulated through selective turnover of proteins and inhibition or promotion of protein activity. The ubiquitin-proteasome pathway controls the degradation of most regulatory proteins, and E3 ubiquitin ligase determines the substrate specificity and timing of ubiquitination for various substrates [19, 20]. Among E3 ligase, F-box proteins typically recognize and interact specific substrate [21]. Most of the well-studied F-box protein have complex substrate [17, 22, 23]. In general, F-box proteins identify distinctive, short degradation motif in their substrates and this interaction between well-known F-box protein and substrate provoke a posttranslational modification [17, 21]. This F-box protein binding typically accessed on their motif, which can controlled through many regulatory mechanisms including post-translational modifications [24]. Also, F-box protein is able to labile protein stability of transcription factors and are positively degraded through the ubiquitin proteasome pathway [20]. This facts consider that protein stability have can be recognize and ubiqutilate by E3 ligase such as F-box protein was interacting with their transcriptional activation or repressor domain. Interestingly, Muratani et al reported that the coupling of the transcriptional activity with the protein allowed prevention hyper-activation of transcription factors by E3 ligase [25]. Furthermore, Liu et al. reported that the E3 ligase FBW7 interacts with the KLF5 transactivation domain for its degradation [26]. That is, ubiquitination via E3 ligase plays a role in tumor suppression and its ubiquitination activity may be a novel therapeutic approach for effectively targeting kinases implicated in cancer. All this leads us to suspect that there may be one of critical F-box protein that target the ICAM-1 protein for degradation through interaction with ICAM-1 domain. Herein, we identified the FBXO4 protein though F-box protein screening and examined whether F-box is related to intracellular events as well as the relationship between ICAM-1 and FBXO4. The goal of this study was to determine whether ICAM-1 stability affects metastatic progression and investigate the relationship between ICAM-1 and FBXO4.

## RESULTS

### ERK pathway regulates ICAM-1 stability, which is critical in breast cancer

**Figure 1:**
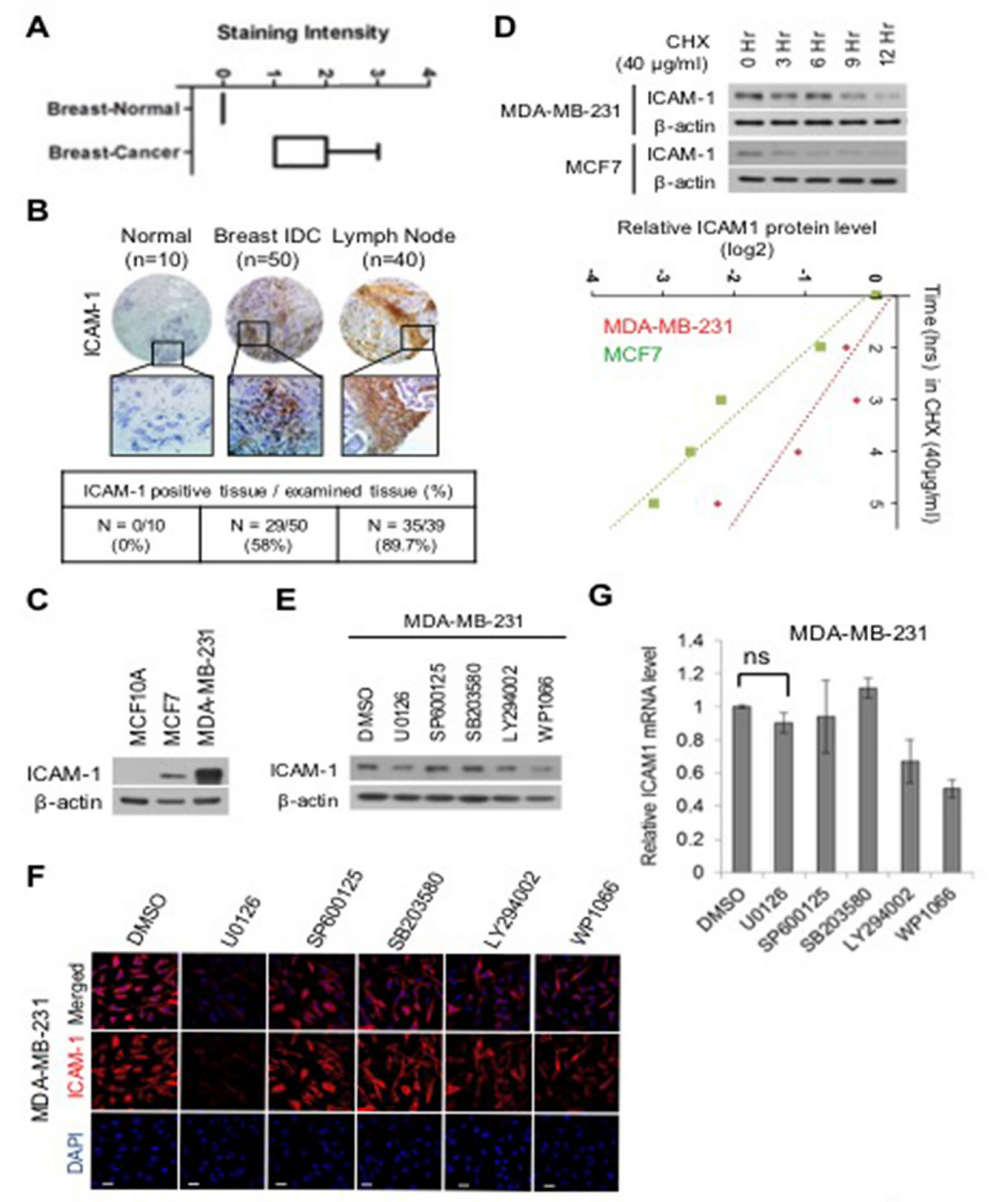

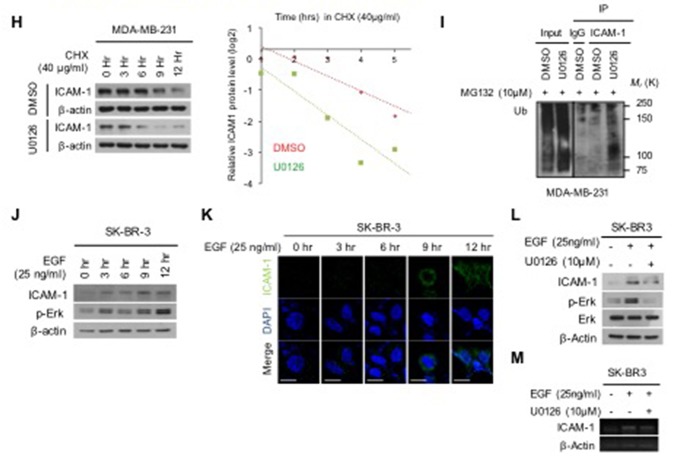
ERK regulates the stability of ICAM-1 in breast cancer **(A)** Analysis of ICAM-1 data from publicly available human target protein databases (http://www.proteinatlas.org/). **(B)** Representative micrographs of human breast cancer tissue. Quantification of ICAM-1 intensity in tissues of normal breast cancer, advanced breast cancer, and metastatic breast cancer. **(C)** Endogenous ICAM-1 in normal epithelial cells, metastatic breast cancer cells, or non-metastatic breast cancer cells was detected by western blot analysis. **(D)** CHX pulse-chase experiments the analysis of the stability of ICAM-1 in metastatic or non-metastatic breast cancer cells that were treated with cycloheximide (CHX, 40 μg /mL). Western blot data were quantified using ImageJ software. **(E-G)** Western blotting (E) ICC (F), and qRT-PCR (G) were performed to detect the influence of the inhibition of various signaling pathways in metastatic breast cancer cells that were incubated for 24 hours. **(H)** Pulse-chase experiments for the analysis of ICAM-1 stability, which was affected by the inhibition of ERK signaling in metastatic or non-metastatic breast cancer cells that were treated with CHX. **(I)** Immunoprecipitation and western blotting for the demonstration of the polyubiquitination of ICAM-1 in metastatic breast cancer cells under ERK inhibition or after the treatment with DMSO and control IgG antibodies for 48 hours. The proteasome inhibitor, MG132 was added to stabilize ubiquitination. **(J)** Relationship of the stability of ICAM-1 with pERK signaling after EGF stimulation in non-metastatic breast cancer cells and SK-BR3 cells was analyzed by western blotting (J) and ICC **(K)**. **(L, M)** Expression levels of ICAM-1 and pERK proteins and mRNAs in non-metastatic breast cancer cells after EGF stimulation and U0126 treatment were analyzed by western blotting (L) and conventional RT-PCR. (M). All western blot data were quantified using ImageJ software. The results are expressed as mean ± SD of three different experiments. (Single asterisks (*) indicate a significant difference (P < 0.05), double asterisks (**) indicate a highly significant difference (P < 0.01), triple asterisks (***) indicated the highest significant difference.)

To determine whether expression of ICAM-1 is altered in different subtypes of breast cancer, we evaluated the level of ICAM-1 in metastatic cancer cells and non-metastatic cells (Figure 1A-1B). As shown in Figure 1A-1B, ICAM-1 level was higher in metastatic breast cancer tissue and lymph nodes than in normal breast cancer. Corresponding to changes in patient tissues, of the 100 breast cancer samples, 10 metastatic cancer cells/tissues were negative and 50 were positive in, whereas 40 progressive breast cancer cells/tissues were strongly positive for ICAM-1. Furthermore, ICAM-1 level was significantly higher in metastatic and advanced cancer cells/tissues (MDA-MB-231) than in normal epithelial cells (MCF10A) (Figure 1B-1C). To confirm that the ICAM-1 is post-translationally controlled in metastatic and non-metastatic breast cancer cells, we first blocked protein synthesis using cycloheximide (CHX) (Figure 1D). We observed that metastatic breast cancer cells (MDA-MB-231) showed consistently decreased ICAM-1 stability compared to that in non-metastatic cancer cells (MCF7). To identify the signaling molecules involved in regulating ICAM-1 stability, we examined changes in ICAM-1 levels following inhibition of different known regulators of its synthetic pathway by western blot analysis, immunocytochemistry (ICC), and quantitative polymerase chain reaction (qPCR) (Figure 1E-1G). WP1066 is a well-known STAT3 signaling inhibitor and recently Kesanakurti et al. also confirmed that STAT3 regulates ICAM-1 expression in glioma [27]. However, as shown in Figure 1E-1G, we confirmed that the level of ICAM-1 protein decreased following treatment with the well-known extracellular signal-related kinase (ERK) inhibitor U0126, whereas its mRNA level was not different as compared to control samples. This result indicated that ICAM-1 stability is controlled at the protein level and not at the transcriptional level by the ERK pathway. The mitogen-activated protein kinase ERK pathway is among the most important pathways involved in cell growth and maturation [28, 29]. It has been suggested that the ERK signaling pathway regulates proliferation and epithelial-mesenchymal transition (EMT) of various cancer cell types. We next evaluated the half-life of ICAM-1 to determine its stability by blocking *de novo* protein synthesis with CHX (Figure 1H). Additionally, to confirm whether ICAM-1 is post-translationally regulated by ubiquitin-mediated proteasomal degradation in metastatic breast cancer, we blocked proteasome activity by MG132 treatment for 6 h in MDA-MB-231 cells and performed an ubiquitylation assay (Figure 1I). Polyubiquitylation of ICAM-1 was significantly higher under ERK inhibition than following dimethyl sulfoxide treatment. Next, to assess whether expression of ICAM-1 changes with cancer progression, the inhibitory effect of ICAM-1 in metastatic or non-metastatic cancer cells was investigated. To determine the role of ICAM-1 in migration and invasion, we blocked endogenous ICAM-1 expression by RNAi. To evaluate the characteristics of ICAM-1 expressing cells, we assessed the migratory and invasive characteristics of metastatic and non-metastatic breast cancer cells by analyzing EMT marker expression and invasion and migration assay in (Supplementary Figure 1A-1D). As shown in Supplementary Figure 1A, knockdown of ICAM-1 evidently decreased in vimentin and ZEB, regulators of EMT, were significantly decreased in metastatic breast cancer. While, overexpressing of ICAM-1 were increased the expression of vimentin and ZEB in non-metastatic breast cancer cells (Supplementary Figure 1B). Supplementary Figure 1C show that inhibition of ICAM-1 decreased the migratory and invasive activities of cells and that blocking of ICAM-1 expression was associated with tumor progression. To determine whether downregulation of ICAM-1 decreases metastasis *in vivo*, metastatic cancer cells, MDA-MB-231-LM1, were transplanted into the tail vein of NOD/SCID mice (Supplementary Figure 1E). As shown in Supplementary Figure 1E, lung metastasis was remarkably decreased following knockdown of ICAM-1 compared to that in controls, suggesting that ICAM-1 is critical for metastatic potential in breast cancer cells. RNAi-mediated removal of ICAM-1 knockdown treatments conducted for 48 h led to alterations in the expression of EMT and regulatory markers, including significant decreases in ICAM-1, vimentin, and Zeb-1. In contrast, ICAM-1 exerted a stimulatory effect on the EMT of the non-metastatic breast cancer cells (Supplementary Figure 1A-1B). These results suggest that high ICAM-1 expression is involved in tumor progression and metastatic potential, and downregulation of ICAM-1 decreases cellular mobility in progressive breast cancer cells. Epidermal growth factor (EGF) stimulation test was performed to determine whether ICAM-1 expression was activated in non-metastatic breast cancer cell. Treatment with 25 ng/mL EGF for 0, 3, 6, 9, or 12 h produced a treatment time-dependent increase in ICAM-1 and pERK expression (Figure 1J-1K). To further confirm the stability of ICAM-1 and its changes levels observed in non-metastatic breast cancer cells conducted under the same conditions, cells treated by EGF and U0126 were analyzed by western blotting, and reverse transcription (RT)-PCR (Figure 1L-1M). As shown in Figure 1J, stimulation by EGF increased ICAM-1 stability and ERK phosphorylation, whereas the increased expression of ICAM-1 and pERK was attenuated by treatment with the ERK inhibitor U0126 (Figure 1L-1M); however, transcript levels were not changed. These results suggest that the ERK pathway was involved in maintaining ICAM-1 stability and that ICAM-1 transcription was not affected in progressive breast cancer or normal cells. That is, ICAM-1 stability and cancer progression appear to be modulated by the post-translational regulation of ICAM-1, and not transcriptional regulation.

### A novel finding of E3 ligase FBXO4 specific-interaction with ICAM-1 in breast cancer cells

**Figure 2:**
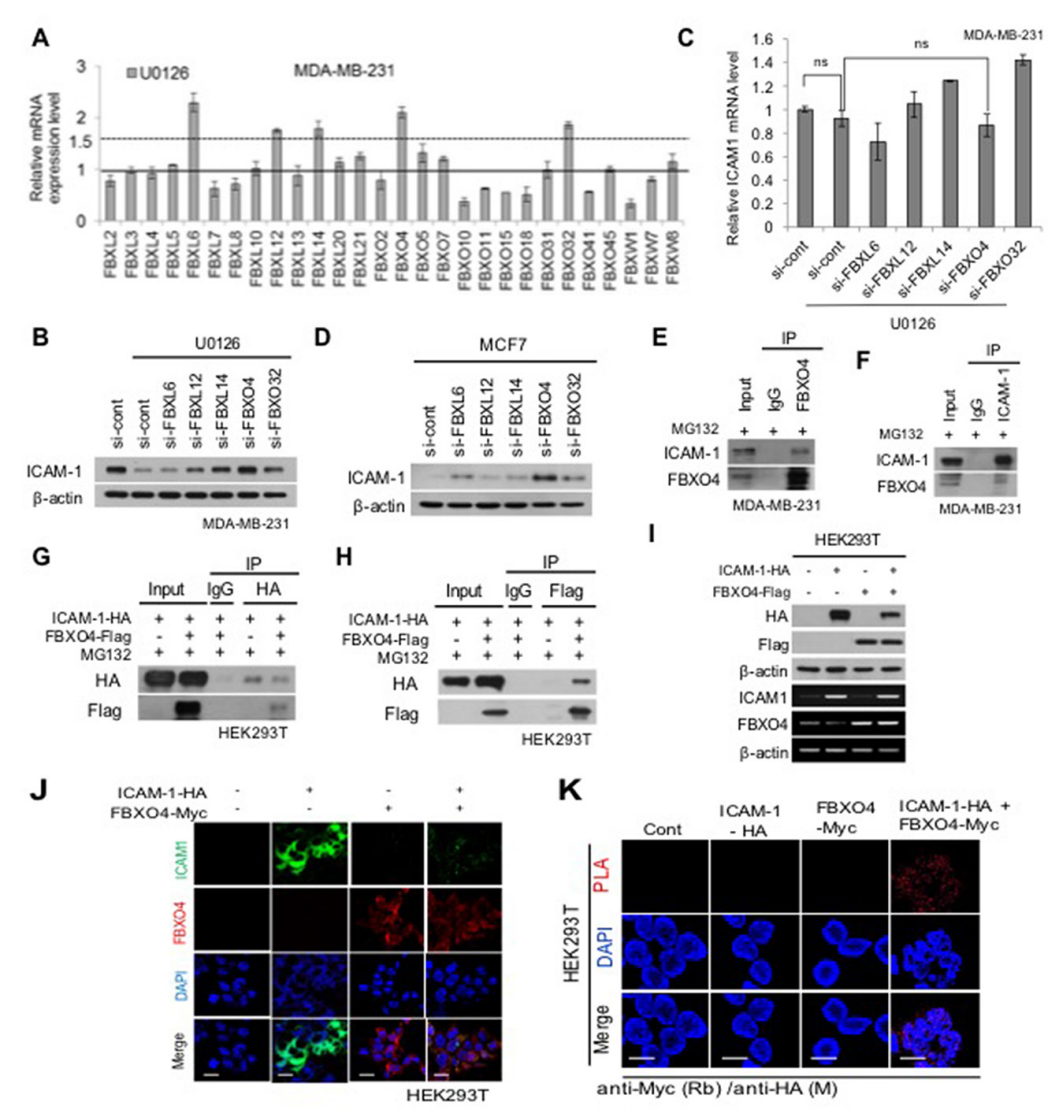

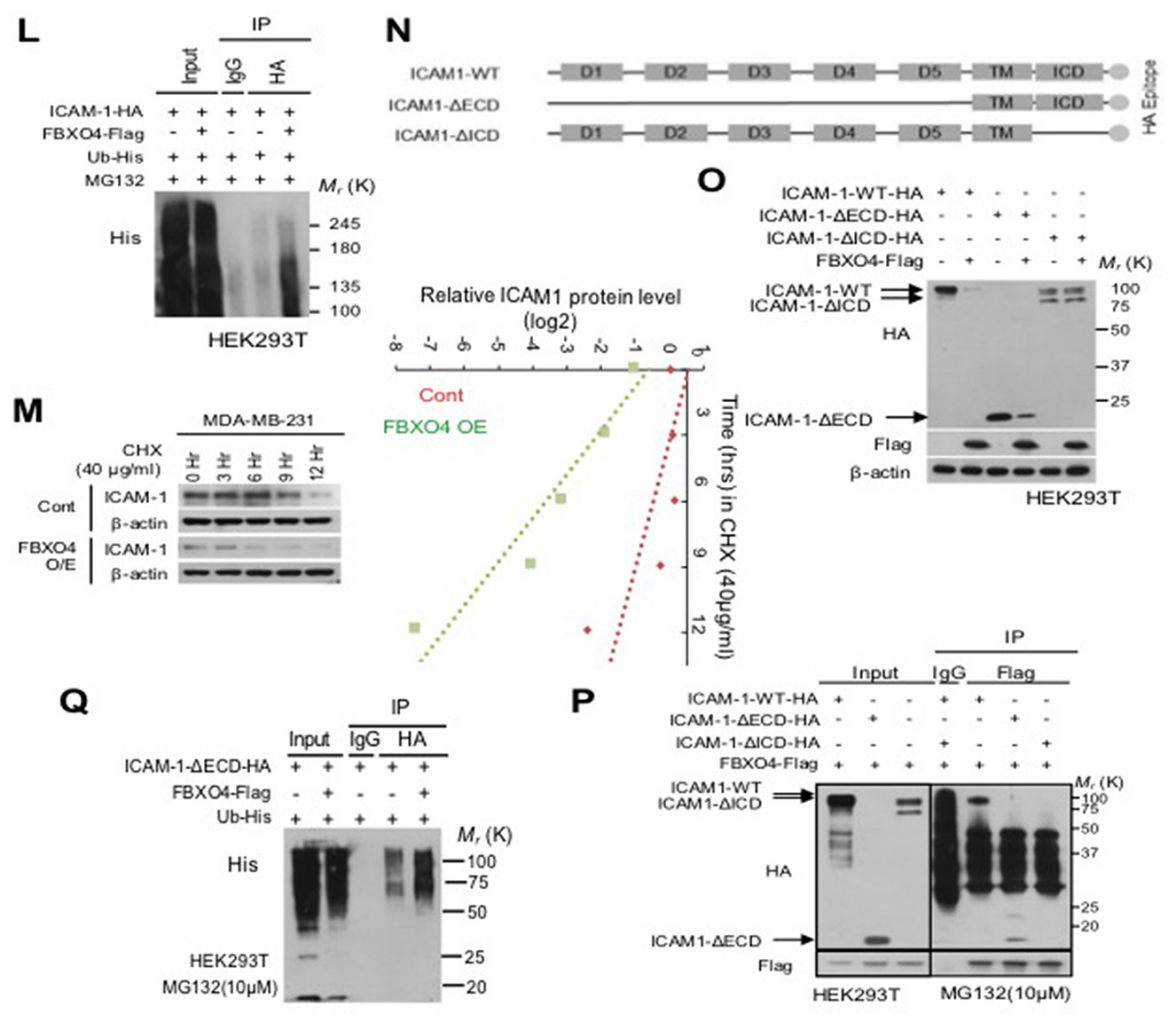
FBXO4, which is an E3 ligase, reduces the stability of ICAM-1 in breast cancer cells **(A)** qRT-PCR was performed to detect the changes in the level of F-BOX mRNA expression in metastatic breast cancer cells corresponding to U0126 treatment. **(B, C)** Western blotting of selected F-BOX candidates was confirmed to detect changes in the stability of ICAM-1 after U0126 treatment, which caused the knockdown of F-BOX protein in metastatic breast cancer cells (B) and analyzed by qRT-PCR under the same condition (C). **(D)** Western blotting for the analysis of changes in ICAM-1 expression due to the knockdown of selected F-BOX candidates in non-metastatic breast cancer cells. **(E, F)** Immunoprecipitation and western blot analysis demonstrated the interaction between ICAM-1 and FBXO4. **(G-H)** For additional confirmation, HEK293T cells that were transfected with ICAM-1-HA or FBXO4-Flag for 48 hours were examined. Cell lysate was collected after MG132 treatment for 6 hours before subjecting to immunoprecipitation and western blot analysis. **(I)** Comparison of the level of transcriptional or post-transcriptional expression that was confirmed in HEK293T cells using ICAM-1-HA and FBXO4-Flag was performed using western blotting and conventional PCR. **(J)** Immunofluorescence images of HEK293T cells with the overexpression or knockdown of ICAM-1-HA alone or together with FBXO4-Myc. Scale bar = 100 μm. **(K)** Binding affinity of FBXO4 and ICAM-1 was assessed by in situ PLA with HEK293T cells, which were transfected with ICAM-1-HA or FBXO4-Myc. **(L)** HEK293T cells were transfected as described in the panel labels. Immunoprecipitation and western blotting were performed using either an anti-HA antibody for pooling HA-tagged ICAM-1 or an anti-Flag antibody against FBXO4-Flag after MG132 treatment. **(M)** CHX pulse-chase assay for ICAM-1 stability degradation by FBXO4 detected by western blotting. **(N)** Schematic of ICAM-1 structure and the deletion mutant construct, which was generated for mapping the interaction domain of ICAM-1-FBXO4. **(O)** Protein expression of WT and ICAM-1-deletion construct by FBXO4, HEK293T cells, which were co-transfected with ICAM-1-WT-HA, ICAM-1-∆ECD, ICAM-1-∆ICD, and FBXO4-Flag, were analyzed by western blotting. **(P)** Wild-type HA-tagged ICAM-1-deletion construct (ICAM-1-∆ECD or ICAM-1-∆ICD) were co-transfected with FBXO4-Flag. Cell lysate was collected after MG132 treatment before subjecting to immunoprecipitation and western blot analysis. **(Q)** Ubiquitination was analyzed as follows. HEK293T cells were co-transfected with ICAM-1-∆ECD-HA and RBXO4-Flag for 48 hours. The cell lysate was treated with MG132 before subjecting to co-IP. All western blot data were quantified using ImageJ software. Also, the IP and co-IP fractions were then subjected to western blot analysis with mouse anti-HA Ab or mouse anti-Flag Ab (co-IP). (*P < 0.05, **P < 0.01 or ***P<0.001.)

Ubiquitin-mediated proteasomal degradation regulates the selective turnover of proteins such as ICAM-1. As demonstrated previously [17, 30], we also observed that the proteasome inhibitor MG132 blocks the degradation of ICAM-1. We hypothesized that ICAM-1 was degraded by the ubiquitinating protein E3 ligase. To identify the proteins involved in ubiquitin-mediated degradation, we focused on the F-box protein. F-box protein is an E3 ligase associated with cellular functions such as signal transduction and cell cycle regulation. We evaluated whether F-box protein regulates ICAM-1. To determine whether E3 ligase is specific for ICAM-1, we analyzed E3 ligase in whole F-box protein, and then selected five F-box proteins: FBXL6, FBXL12, FBXL14, FBXL32, and FBXO4. Selected candidates were screened by qPCR (Figure 2A). First, to investigate the relationship between ICAM-1 and E3 ligase, ICAM-1 levels were measured by western blotting following blockade with U0126 and downstream signals were determined (Figure 2B-2D). Among F-box proteins, inhibition of FBXO4 clearly increased expression of ICAM-1 in MDA-MB-231 and MCF7 cells (Figure 2B-2D). In contrast, to examine whether the expression of the selected proteins recovered in MDA-MB-231 cells, we measured the changes in the siRNA-mediated inhibition of F-box protein (Figure 2D). As shown in Figure 2C, ICAM-1 mRNA expression was not remarkably decreased following knockdown of FBXO4 by U0126 compared to the expression observed following knockdown of other selected F-box proteins. We next examined the interaction between FBXO4 and ICAM-1. Ubiquitylation occurs via a sequence of enzymatic events. To determine whether FBXO4 degrades ICAM-1 by specific ubiquitination, the ICAM-1 and FBXO4 interaction was tested in metastatic breast cancer cells by co-immunoprecipitation (IP) and western blot analysis following treatment with a proteasome inhibitor (Figure 2E-2F); the results were confirmed in HEK293T cells (Figure 2G-2H). Additionally, we performed simultaneous observation of the interactions of between ICAM-1 and FBXO4, we carried out the in situ proximity ligation assay (PLA). We confirmed that the absence of the signals in ICAM-1-HA and FBXO4-Myc each, while the in situ PLA signals between ICAM-1-HA and FBXO4-Myc were clearly observed due to their interaction after MG132 treatment (Figure 2I). Furthermore, western blotting, conventional RT-PCR, and ICC experiments confirmed FBXO4 regulates ICAM-1 (Figure 2J-2K). We also found that FBXO4 controlled ICAM-1 stability at the post-translational level, but not the post-transcriptional level, in HEK293T cells. To further investigate whether FBXO4 ubiquitylates ICAM-1, we co-transfected HEK293T cells with ICAM-1-HA, FBXO4-Flag, and His-ubiquitin and treated the cells with MG132 for 6 h to prevent protein degradation before conducting a ubiquitination assay (Figure 2L). As shown in Figure 2L, a clear increase in ubiquitin-mediated degradation of the ICAM-1-HA/FBXO4-Flag complex was observed. Consequently, the interaction of FBXO4 and ICAM-1, indicating the binding specificity of FBXO4 to ICAM-1 enhanced FBXO4-mediated ICAM-1 poly-ubiquitination. In protein stability analysis in which *de novo* protein synthesis was blocked with CHX, FBXO4 accelerated ICAM-1 degradation and lowered ICAM-1 stability (Figure 2M). FBXO4 belongs to the largest family of E3 ligases and is a cullin-RING ligase, which requires F-box protein for substrate recognition [19, 20, 27]. It was unclear which ICAM-1 domain is ubiquitinated after being recognized by FBXO4. We assumed that ICAM-1 protein must be ubiquitinated after being recognized by FBXO4. To test this hypothesis, we used a modified ICAM-1 lacking the membrane domain (ICAM-1-∆ECD; ICAM-1-deletion of extracellular domain) and intracellular domain (ICAM-1-∆ICD; ICAM-1-deletion of intracellular domain) and transfected HEK293T cells before western blotting and co-IP analysis (Figure 2N). ICAM-1 pulled down together with FBXO4 protein, confirming that the interaction between ICAM-1 and FBXO4 occurred in the intracellular region before ubiquitination (Figure 2O-2P). As shown in Figure 2P, ICAM-1-∆ICD-HA and FBXO4-Flag did not bind to each other, indicating that the ICAM-1 interaction is specific to the intracellular domain region and that this interaction may be critical for the FBXO4-ICAM-1 interaction that leads to the ubiquitin-mediated proteasomal degradation of ICAM-1 (Figure 2Q). We demonstrated that the stability of ICAM-1 decreases via FBXO4-mediated ubiquitination, which marks the molecule for proteasomal degradation.

### FBXO4 regulates EMT and metastasis of breast cancer

**Figure 3:**
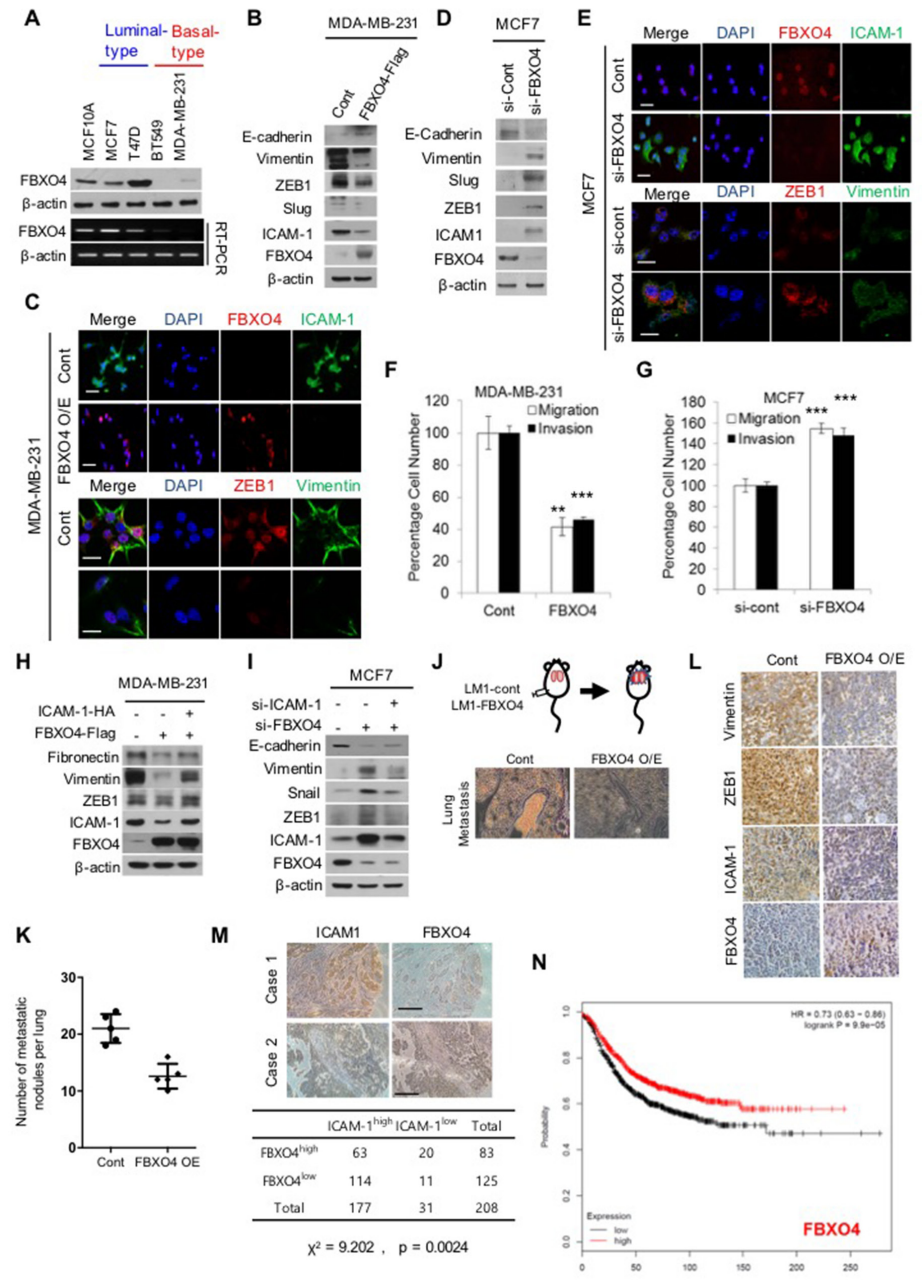
FBXO4 regulates EMT and metastasis in breast cancer **(A)** Expression of the *FBXO4* gene between the luminal (metastatic) and basal subtypes (non-metastatic) of breast cancer was detected by conventional RT-PCR. **(B, C)** Levels of the EMT-related proteins, vimentin, SLUG, ICAM-1, and FBXO4 in FBXO4-overexpressing metastatic (B) or FBXO-knockdown non-metastatic breast cancer cells (C) were detected by western blot analysis. β-actin served as a loading control. **(D, E)** Immunofluorescence for analyzing the expression of ICAM-1, FBXO4, ZEB1, and vimentin in FBXO4-overexpressing metastatic breast cancer cells (D) or FBXO-knockdown non-metastatic breast cancer cells (E). Scale bar = 10 μm. **(F, G)** Invasion and migration of metastatic or non-metastatic cancer cells with the overexpression (F) or knockdown (G) of FBXO4. **(H, I)** Levels of protein expression in metastatic (H) or non-metastatic breast cancer cells (I) after the overexpression or knockdown of FBXO4 alone or together with ICAM-1. β-actin served as a loading control. **(J-L)** Transplantation of control MDA-MB-231 LM-1 or FBXO4-overexpressing LM-1 cells into fat pads of mice (n=5). The mice were sacrificed 4 weeks later. Representative images of lung metastasis, which were detected by H&E staining. (K) Number of metastatic foci in the lung that was counted for EBXO4-positive cells. (L) Bright-field images of the primary tumor tissue that was derived from an *in vivo* experiment, wherein it was stained with the EMT-related markers, ICAM-1 and FBXO4, and analyzed by IHC. Advanced breast cancer cells served as control. **(M)** Representative images of the expression of the FBXO4 and ICAM-1 proteins as determined by IHC of the clinical specimen. Normal breast tissue served as control (at 200× magnification). **(N)** Kaplan-Meier plot of patients who survived metastasis stratified by FBXO4 expression. Data were obtained from the breast cancer dataset from Oncomine. All data are expressed as the mean of data collected from three independent experiments.(*P < 0.05,**P < 0.01 or ***P<0.001.)

To determine the expression and effects of altered ICAM-1 stability on EMT, we measured the post-transcriptional RNA and protein levels of ICAM-1 together with those of EMT markers by RT-PCR and western blotting, and observed clear differences in various subtypes of breast cancer cells (Figure 3A-3B and 3D). As shown in Figure 3A, the transcriptional level of FBXO4 was significantly higher in the luminal subtype breast cancer cell than in the basal subtype. Additionally, the basal subtype of breast cancer has been associated with aggressive metastatic cancer and poor prognosis [2, 10]. Therefore, to investigate whether ICAM-1 stability is associated with the mesenchymal movement and invasiveness of metastatic breast cancer cells, we assessed the migratory and invasive characteristics of metastatic and non-metastatic breast cancer cells by analyzing EMT marker expression. As shown in Figure 3B-3C, the protein expression of vimentin and ZEB, regulators of EMT, were significantly decreased in metastatic cancer. In contrast, knockdown of FBXO4 dramatically increased the expression of ICAM-1 and markers of EMT (Figure 3D-3E). Interestingly, overexpression of FBXO4 decreased the migration and invasive ability of metastatic cancer cells, while knockdown of FBXO4 markedly promoted the migratory and invasive properties in non-metastatic cancer cells in the recovery experiments (Figure 3F-3G and Supplementary Figure 2A). These results indicate that FBXO4 deregulates EMT processes, such as migration and invasion, in metastatic cancer cells. FBXO4 overexpression decreased EMT in metastatic breast cancer cells with a loss of fibronectin, vimentin, and ZEB1 (Figure 3H). In metastatic breast cancer cells co-expressing ICAM-1 and FBXO4, the level of ICAM-1 was significantly higher than that in cells overexpressing only FBXO4. The increase in ICAM-1 level reversed the increase in EMT-related markers (Figure 3H). Additionally, we confirmed that knockdown of FBXO4 alone or co-knockdown of FBXO4 and ICAM-1 reversed these effects in non-metastatic breast cancer cells (Figure 3I). Based on these results, to investigate whether FBXO4 overexpression affects metastasis *in vivo*, a total of 1 × 10^6^ stable cells over-expressing FBXO4 were injected into the fat pad of NOD/SCID mice (Figure 3J-3L). After 4 weeks, we observed that tumor size and weight were decreased in FBXO4 overexpressing group (Supplementary Figure 2B-2C). Additionally, as shown in Figure 3J-3N, FBXO4 overexpression significantly reduced tumor metastasis *in vivo*. Kaplan-Meier survival analysis of 500 cases obtained from Oncomine showed that breast cancer patients with high FBXO4 expression had longer survival times than those with low FBXO4 expression (P = 0.00001, Figure 3N) [31]. Furthermore, immunohistological analysis confirmed an inverse relationship between the expression of FBXO4 and ICAM-1 (Figure 3L). As expected, the expression of ICAM-1 clearly augmented the level of EMT and metastasis markers, while expression of FBXO4 disrupted the effects of ICAM-1 on metastasis and decreased the level of EMT regulators such as vimentin, and ZEB1. Besides, we confirmed that high expression of ICAM-1 was observed in human metastatic breast cancer tissue whereas expression of FBXO4 was decreased particularly (Figure 3M). This finding suggests that FBXO4 and ICAM-1 have a negative co-relationship, and FBXO4 can suppress tumor progression by inhibiting the EMT-promoting effect of ICAM-1. These results indicate that the expression of FBXO4 in breast cancer patients is associated with positive clinical outcomes and may be explored as a tumor suppressive strategy.

### ERK-induced miR340 regulates FBXO4

**Figure 4:**
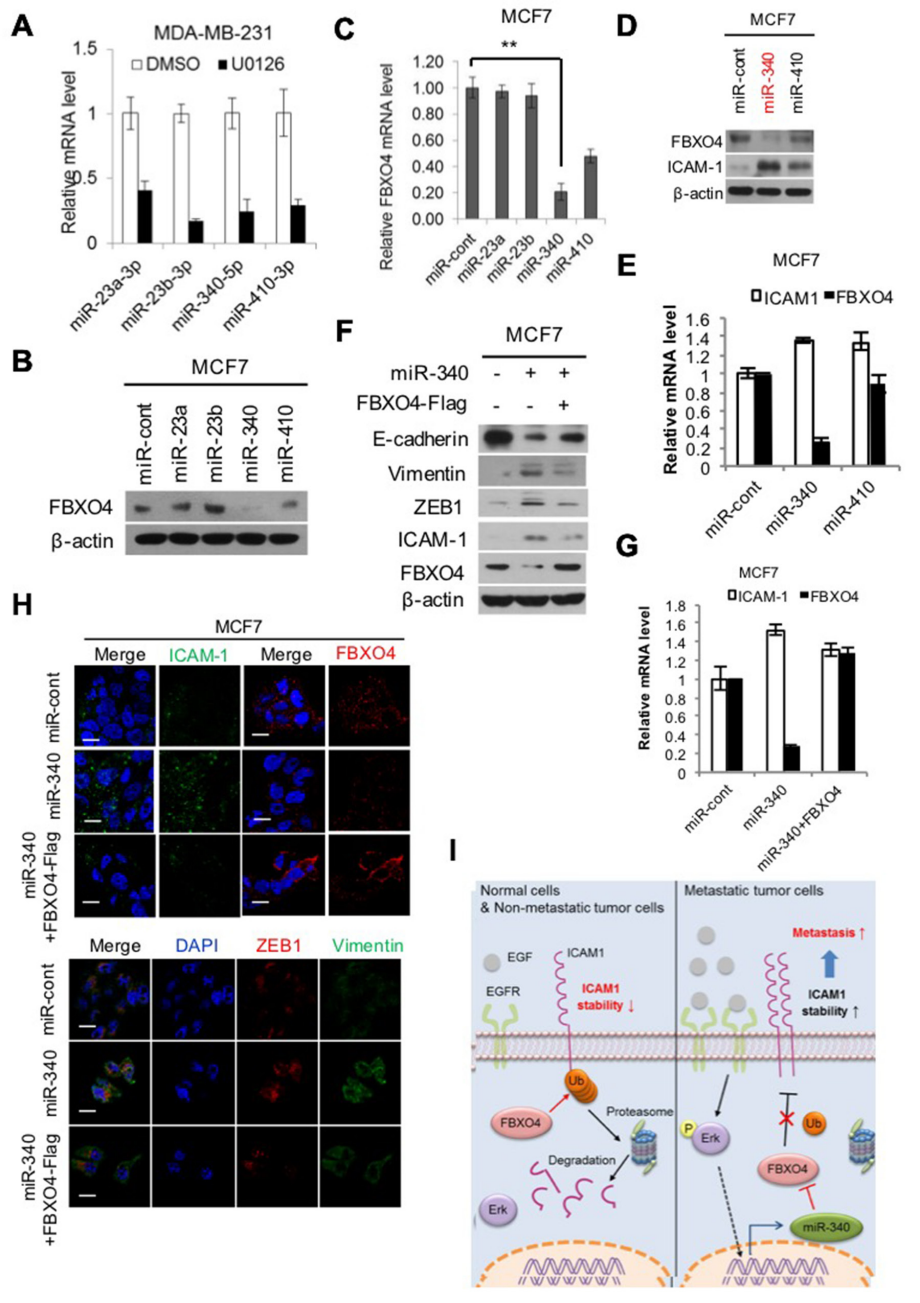
miR-340, which is induced by ERK, regulates FBXO4 **(A, B)** Analysis of miRNA data from publicly available databases for miRNA target prediction; miRNA.org (http://www.microrna.org/microrna/home.do). (A) Level of miRNA expression in metastatic breast cancer corresponding to U0126 treatment was analyzed by qRT-PCR. **(B, C)** Expression levels of FBXO4 protein and mRNA in non-metastatic breast cancer cells that were transfected with selected miRNA candidates were analyzed by western blotting and qRT-PCR. **(D, E)** Expression levels of FBXO4 and ICAM-1 protein and mRNA after the transfection of non-metastatic breast cancer cells with miR340 or miR410 were detected by western blotting (D) and qRT-PCR (E). **(F and H)** Representative immunofluorescence image of the expression levels of ICAM-1, FBXO4, and EMT-related marker proteins in response to miR340 with or without FBXO4, which was analyzed by western blotting (F) and ICC (H). **(G)** Levels of ICAM-1 and FBXO4 mRNA expression in non-metastatic breast cancer cells that were transfected with miR340 alone or together with FBXO4. The results of the mRNA experiments were normalized to the mRNA level of β-actin. **(I)** Schematic model of regulation of ICAM-1 stabilization dependent corresponding to FBXO4 in tumor cells. (*P < 0.05, **P < 0.01 or ***P<0.001.)

To elucidate which miRNA regulates FBXO4 in the ERK signaling pathway, we performed a literature search of ERK signal-related genes and their conserved miRNAs using the Miranda program. Four miRNAs were found by prediction analysis, all of which were downregulated by the ERK inhibitor U0126 in metastatic or non-metastatic breast cancer cells (Figure 4A); particularly, the level of FBXO4 was significantly decreased in cells transfected with miR340 (Figure 4B). As expected, we found that FBXO4 is regulated by ERK signaling via miR340 and the mRNA level of miR340 was the lowest of all other predicted miRNAs (Figure 4C). Additionally, the level of ICAM-1 was elevated when FBXO4 was downregulated by miR340, while upregulation of FBXO4 by miR410 decreased the expression of ICAM-1 in MCF7 cells (Figure 4D-4E). EMT is an important mechanism in cancer progression. We detected the levels of EMT regulatory factors and markers, including E-cadherin, vimentin, ZEB, ICAM-1, and FBXO4, following knockdown of specific miRNAs in cells overexpressing FBXO4. As shown in Figure 4F, overexpression of miR340 resulted in a large increase in EMT-related regulatory molecules and markers such as E-cad, vimentin, and ZEB1 as well as ICAM-1 expression, while overexpression of FBXO4 restored FBXO4 levels and EMT markers. We obtained identical results in quantitative PCR and ICC in cells co-transfected with miR340 and FBXO4 (Figure 4G-4H). These results indicate that miR340 was a major factor in the downregulation of FBXO4 and miR340 disrupted tumor suppressive effect, similarly to the ERK-mediated inhibition of FBXO4. Notably, activation of FBXO4 deregulated EMT and tumor progression, including processes such as cell migration and invasion, and FBXO4 regulated ICAM-1 stability via the ERK pathway. Taken together, to show that regulation of ICAM-1 stabilization dependent corresponding to FBXO4, we summarized a graphical scheme (Figure 4I). In normal cells and non-metastatic tumors, E3 ligase FBXO4 is highly expressed. FBXO4 mediates poly-ubiquitination of its substrate ICAM-1, and this interaction leads to the ubiquitin-mediated proteasomal degradation of ICAM-1. However, the ERK pathway is highly activated in metastatic tumors. Activated ERK promotes the expression of miR-340, which downregulates FBXO4. Therefore, the stability of ICAM-1 is increased in metastatic tumors via the ERK pathway-mediated downregulation of FBXO4. Increased ICAM-1 stability results in increased metastasis and invasiveness of cancer cells.

## DISCUSSION

In this study, we found that ICAM-1 is involved in breast cancer metastasis and that its stability is regulated via the ERK pathway. Increased ICAM-1 expression increased metastatic progression potential in the luminal subtype of breast cancer compared with that in normal epithelial tissues. ICAM-1 expression may enable malignant progression via ERK signaling, which likely regulates cancer progression by modulating ICAM-1 stability. The present study showed that ICAM-1 is degraded by the ubiquitin proteasome pathway. Control of protein stability via the ubiquitin-mediated proteasomal degradation pathway plays an important role in regulating cellular processes, as many regulatory proteins are involved in these processes. FBXO4 showed specificity for ICAM-1 and acts as a tumor suppressor for ICAM-1 mediated progressive breast cancer. The proteasome inhibitor MG132 reduced the effects of ICAM-1, suggesting that ICAM-1 levels are decreased by proteasomal degradation. ICAM-1 and FBXO4 showed a strong negative correlation in metastatic breast cancer. More recently, different E3 ligases, F-box proteins, have been reported to be widely regulated or inhibited in malignant tumor progression, suggesting roles as tumor suppressors [17, 20, 32, 33]. FBXO4, an F-box protein with numerous substrates in biologically diverse signaling pathways, has key functions in cancer progression and may be a promising therapeutic target. We also investigated five suitable FBOX protein candidates involved in the ERK pathway using the ERK inhibitor U0126. Notably, FBXO4 shows specificity towards ICAM-1 and may have different ubiquitin-dependent regulatory roles such as the suppression of metastatic cancer progression via diverse pathways. Duan et al. suggested that FBXO11 is a tumor suppressor that functions via BCL6 degradation in B cells, but no structural information is available for FBXO11 [32]. Despite the lack of molecular information, F-box proteins remain attractive potential targets for tumor therapies. The ubiquitin-mediated proteasomal degradation system regulates the turnover of proteins that control various cellular events, such as cell cycle progression, development, transcriptional activity, and metabolic regulatory steps [33–36]. Moreover, we found that the E3 ligase FBXO4 decreases ICAM-1 stability and EMT of metastatic breast cancer cells. FBXO4 protein affects the activation or inhibition of downstream signaling in metastatic breast cancer cells. Alternatively, specific binding of the proteins to the substrates may lead to the activation of other regulatory mechanisms that ultimately activate the proteasome for specific degradation of the substrate. FBXO4 bound to the intracellular domain of ICAM-1 and its binding promoted the ubiquitin-mediated proteasomal degradation of ICAM-1. FBXO4 plays an important role in regulating many cellular processes and its expression is regulated by various proteins. EMT is an important process in tumor metastasis and is involved in the loss of epithelial phenotypes [37], which have been linked to the Wnt [38], Notch [39], MAPK/ERK [40], and various EMT pathways. We elucidated two anti-tumorigenic mechanisms of E3 ligase FBXO4: reduction of ICAM-1 stability and suppression of cancer cell migration and invasion. In the present study, we observed high ICAM-1 expression in advanced or metastatic breast cancer cells and found that ICAM-1 regulated EMT. These findings suggest that this protein may be involved in disease progression in breast cancer. Particularly, ICAM-1 expression clearly enhanced the expression of genes known to regulate EMT and metastatic processes, while expression of FBXO4 lowered ICAM-1 stability and promoted metastasis. Thus, FBXO4 decreases EMT *in vivo* and *in vitro* and may have a major tumor suppressive role in metastatic breast cancer.

Huang et al. [41] reported that miR340 is associated with metastasis by targeting specific pathway, including non-small-cell lung cancer [42], breast cancer [43], and osteosarcoma [44], and suppresses tumor cell migration and invasion. In the present study, we found that miR340 is a potential tumor suppressor associated with FBXO4. Our results demonstrate that miR340 significantly downregulated FBXO4 and overexpression of miR340 increased EMT in FBXO4-downregulated MCF7 cells. Tumor progression and tumor growth were also affected by the transcriptional modulation of FBXO4. Although miR340 appears to be downregulated in various types of cancers, its role and underlying mechanism of action in the ubiquitin-mediated degradation of FBXO4 remained unclear. Therefore, we examined the relationship between FBXO4 and ICAM-1 as well as the suppressive role of ICAM-1 in the regulation of metastatic progression, which was dependent on FBXO4, in metastatic breast cancer cells. As the specificity of ubiquitin-mediated proteasomal degradation is tightly controlled by E3 ligase FBXO4, which targets its specific-present substrate protein for degradation, it is very important to determine the mechanism of ICAM-1 stabilization by FBXO4, which controls ICAM-1 ubiquitination and degradation. Our findings showed that FBXO4 is a strong regulator of ubiquitination and maintains the stability of ICAM-1 during ubiquitin-mediated proteasomal degradation.

## MATERIALS AND METHODS

### Cell culture

MDA-MB-231, MCF7, MCF10A, and SK-BR3 human breast cancer cell lines and HEK293T cells, were purchased from American Type Culture Collection (Manassas, VA, USA), and LM1 cells were obtained from lung metastases of mice injected with MDA-MB-231 cells were maintained at 37°C with 5% CO_2_ in Dulbecco’s modified Eagle medium (DMEM) supplemented with 10% fetal bovine serum (FBS), penicillin (100 U/mL), and streptomycin (100 μg/mL). MCF10A cells were cultured in DMEM/F12 media, supplemented with horse serum (5%, PAA), hydrocortisone (0.5 μg/ml), insulin (10 μg/ml), cholera toxin (0.1 μg/ml) and EGF (2 ng/ml). All medium and supplements were purchased from GIBCO (Grand Island, NY, USA).

### Transfection

ICAM-1-HA (Addgene), FBXO4-Flag (origene), and Ub-His were transfected into cells using Lipofectamine and Plus reagents (Invitrogen, Carlsbad, CA, USA) according to the manufacturer’s protocol. To transfect siRNAs or miRNAs, Lipofectamine 2000 (Invitrogen) was used according to manufacturer’s protocol. All siRNAs and miRNAs were purchased from Genolution Phamaceuticals, Inc. (Seoul, Korea). Ub-His plasmid was kindly gifted by Seunghee Bae (Konkuk University).

### Chemical reagents and antibodies

Chemical reagents cycloheximide (40 μg/mL), MG132, proteasome inhibitor (10 μM), U0126, ERK inhibitor (10 μM), SP600125, JNK inhibitor (10 μM), SB203580, P38 inhibitor (10 μM), LY294002, PI3K inhibitor (10 μM), and WP1066, STAT3 inhibitor (2 μM) were purchased from Calbiochem (San Diego, CA, USA). Monoclonal antibodies to HA and FBXO4 and polyclonal antibodies to VIM, SLUG, and ubiquitin were purchased from Santa Cruz Biotechnology (Santa Cruz, CA, USA). Polyclonal antibodies to SNAIL, p-ERK, and ERK were purchased from Cell Signaling Technology (Danvers, MA, USA). The monoclonal antibody to the His-Tag was purchased from Abcam (Cambridge, UK). Monoclonal antibodies to β-actin and ZEB1 were obtained from Sigma (St Louis, MO, USA). Anti-rabbit IgG Alexa Fluor 488, anti-mouse IgG Alexa Fluor 488, anti-rabbit IgG Alexa Fluor 590, and anti-mouse IgG Alex Flour 546 were purchased from Invitrogen. Control anti-mouse IgG and anti-rabbit IgG were purchased from Santa Cruz.

### Invasion and migration assay

MDA-MB-231 (2 × 10^4^), LM1 (2 × 10^4^), and MCF7 (6 × 10^4^) cells were seeded on a Boyden chamber (0.8 μm pore size, Corning, Corning, NY, USA). For the invasion assay, the Boyden chambers were pre-coated with growth factor-reduced Matrigel; for the migration assay, the Boyden chambers were uncoated. Medium containing FBS was used to fill the bottom chamber. Invaded and migrated cells were stained with Diff-Quick kit (Thermo Fisher, Waltham, MA, USA) and photographed with an inverted microscope. The number of cells was counted in five microscopic fields from each well and the percentage of invaded and migrated cells was determined relative to control cells.

### Western blot analysis

Cell lysates were extracted from the cell pellet using lysis buffer (40 mM Tris-HCl pH 8.0, 120 mM NaCl, 0.1% Nonidet-P40) supplemented with protease inhibitors. Proteins in whole-cell lysates were separated by SDS-PAGE and transferred to a nitrocellulose membrane (Amersham, Amersham, UK). Nitrocellulose membranes were blocked with 5% skim milk in phosphate-buffered saline containing Tween 20 and incubated with primary antibodies overnight at 4°C. The blots were then incubated with the appropriate horseradish peroxidase (HRP)-conjugated secondary antibodies and proteins were visualized by enhanced chemiluminescence (Amersham), according to the manufacturer’s protocol. Secondary antibodies, anti-mouse IgG-HRP, anti-goat IgG-HRP, and anti-rabbit IgG-HRP were purchased from Santa Cruz Biotechnology.

### Cycloheximide (CHX) pulse chase assay

After culturing overnight, the cells were treated with U0126 or transfected. Two days after transfection or 24 h after U0126 treatment, the cells were treated with 40 μg/mL CHX dissolved in DMSO, and total protein lysates were harvested at each time point and subjected to immunoblotting to analyze the half-life of ICAM-1.

### Immunocytochemistry

Cultured cells were fixed on a cover slip using 4% paraformaldehyde (PFA) and permeabilized with 0.2% NP-40 in PBS. Following fixation, cells were blocked with 5% normal goat serum in PBS and incubated at 4°C overnight with primary antibodies against ICAM-1, FBXO4, Vimentin, and Zeb1 in blocking buffer. Staining of proteins was visualized with anti-rabbit or anti-mouse Alexa Fluor 488 and anti-rabbit or anti-mouse Alexa Fluor 546 secondary antibodies (Molecular Probes, Seoul, Korea). DAPI was used to counterstain the nuclei (Sigma).

### Quantitative reverse transcription polymerase chain reaction (qRT-PCR)

Total cell RNA was isolated using the Trizol reagent (Invitrogen) and cDNA was prepared using Super-Script III (Invitrogen) according to the manufacturer’s instructions. qRT-PCR was performed using the KAPA SYBR FAST qRT-PCR kit (Wilmington, USA) in Rotor Gene Q (Qiagen), and results were analyzed by ΔCt value relative to control sample and expressed as fold changes.

### Co-immunoprecipitation assay

Cell lysates were prepared using the lysis buffer as described in western blot analysis. The lysates were incubated with primary antibodies suitable for immunoprecipitation overnight at 4°C. The mixtures were then incubated with Protein A-Agarose beads (Santa Cruz Biotechnology, Inc.) for 2 h, and the supernatant was collected by centrifugation at 1500 *g*. The protein A-Agarose beads were washed three times with cold PBS and immunoprecipitates were dissolved in SDS and analyzed by western blot.

### In-situ proximity ligation assay (PLA)

Cells cultured on a cover slip were treated with 10 μM MG132 for 6 h and fixed with 4% paraformaldehyde. Fixed cells were permeabilized with 0.2% NP-40 in PBS and blocked with 5% goat serum in PBS. After blocking, the cells were incubated with primary antibodies (anti-Myc rabbit and anti-HA mouse; 1:300 in blocking buffer) at 4°C overnight. *In situ* PLA was performed according to the manufacturer’s protocol using a Duolink Detection Kit with a pair of nucleotide-labeled secondary antibodies. Following ligation and amplification of the PLA probes, the signals were visualized by confocal microscopy.

### Animal experiment

For orthotopic primary tumor formation, LM1 control cells or LM1 FBXO4 over-expressing cells (1 × 10^6^) suspended in 40 μL PBS were injected into the fat pad of 8–10-week-old female NOD/SCID mice (n = 10). Mice were anesthetized and a small incision was made to expose the mammary gland. Primary mammary tumor growth was measured in 4-day intervals after injection. Mice with tumors of representative size and weight in each group were sacrificed at day 28–30 after impanation. Lung metastatic foci were also counted after sacrifice.

### Immunohistochemistry

Mice tissues (lungs and xenografts) were fixed in 4% formalin to prepare paraffin sections. Paraffin-embedded tissue sections were deparaffinized in xylene, 100%, 95%, 70%, and 50% ethanol, and then PBS. Antigens were retrieved using 10 mM citrate buffer. Sections were stained with hematoxylin and eosin or immunostained overnight at 4°C with the primary antibody. After washing in PBS, biotinylated goat anti-rabbit IgG or anti-mouse IgG antibody was applied to the sections for 1 h. After washing in PBS, the ABC reagent (Vector Laboratories Inc., Burlingame, CA, USA) was applied to the sections for 1 h. Color development was performed using 3,3′-diaminobenzidine (Vector Laboratories). After counter-staining with hematoxylin and clearing with a graded ethanol series and xylene, the sections were mounted in Canada balsam. Images were captured with a DP71 digital imaging system on an IX71 microscope (Olympus, Tokyo, Japan).

### Human tissue microarray

Human breast cancer tissue microarray samples were obtained from US Biomax (BR2085c).

### Statistical analysis

All experimental data are reported as the means; error bars represent standard deviation. Statistical analyses were performed using non-parametric Student’s *t*-tests.

## SUPPLEMENTARY MATERIALS FIGURES


