# Supplementary material for: Regulation of FBXO4-mediated ICAM-1 protein stability in metastatic breast cancer
Source: Oncotarget. 2017 Sep 15;8(47):83100–13. doi: 10.18632/oncotarget.20912 (PMC5669953; doi:10.18632/oncotarget.20912)
Supplement: Supplementary file 1 [file oncotarget-08-83100-s001.pdf]

Regulation of FBXO4-mediated ICAM-1 protein stability in metastatic breast cancer

SUPPLEMENTARY MATERIALS

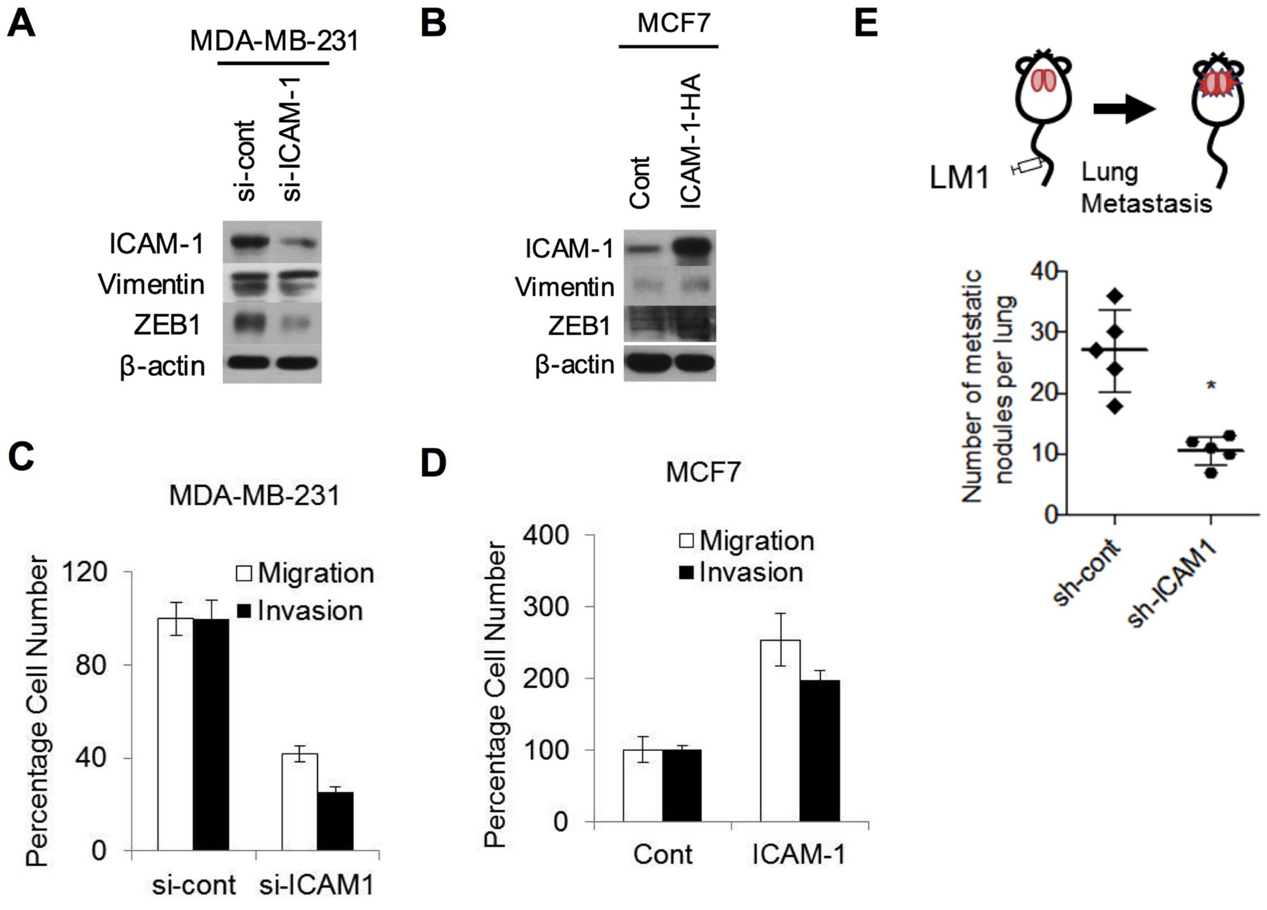

Supplementary Figure 1: ICAM-1 is a critical regulation in metastatic breast cancer. (A) Expression levels of ICAM-1 and the EMT markers (vimentin and ZEB1) in ICAM-1-knockdown metastatic breast cancer cells (A) and ICAM-1-overexpressing non-metastatic breast cancer cells **(B)**. **(C, D)** Invasion and migration of metastatic breast cancer cells with ICAM-1 knockdown or overexpression. **(E)** For lung metastasis, tail veins were injected with 1 x 10^6 of ICAM-1-knockdown metastatic breast cancer cells. The results are expressed as mean ± SD of three different experiments. (*$P < 0.05$, **$P < 0.01$ or ***$P<0.001$.)

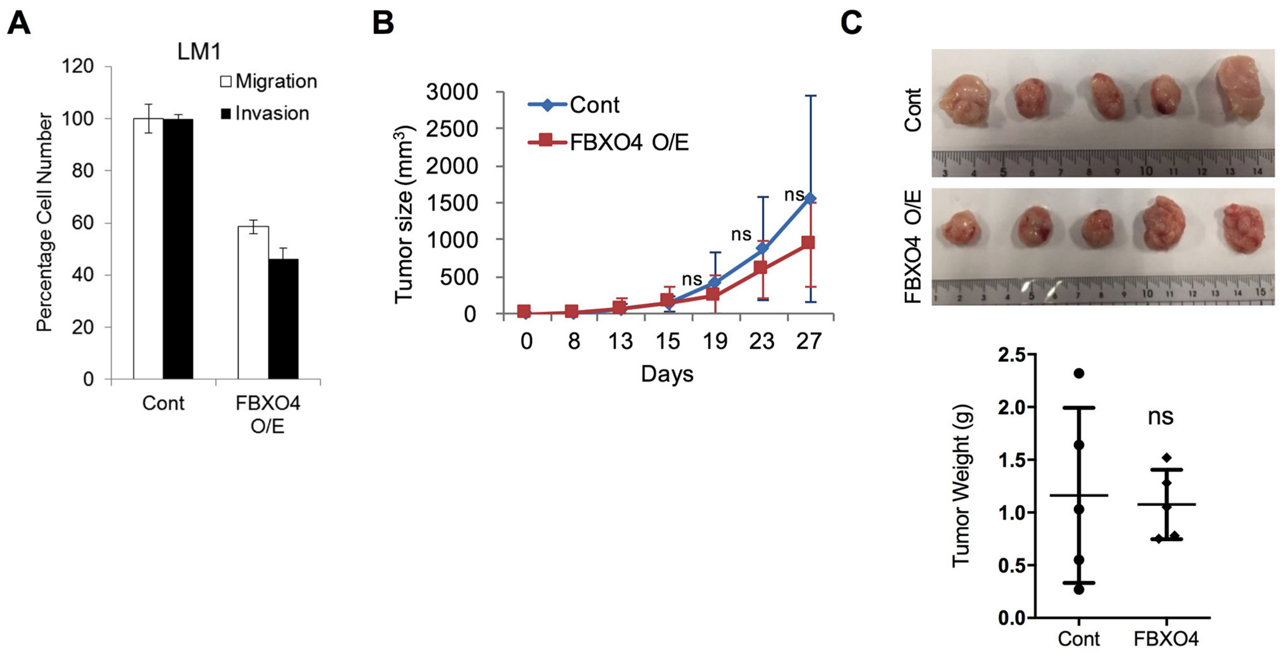

Supplementary Figure 2: (A) Invasion and migration of metastatic breast cancer cells with FBXO4 overexpression. **(B, C)** *In vivo* monitoring of tumorigenicity. 1 x 10^6 of the FBXO4-overexpressing metastatic breast cancer cells were injected into NOD or SCID mice (n=5), and primary breast tumor growth was measured at 4-day intervals after the injection. (C) Histogram represents the comparison of tumor size. Each time point represents the mean ± S.E. of the three xenograft tumors in each group. (*$P < 0.05$, **$P < 0.01$ or ***$P<0.001$.)
